# Aerobic exercise training enhances the *in vivo* cholesterol trafficking from macrophages to the liver independently of changes in the expression of genes involved in lipid flux in macrophages and aorta

**DOI:** 10.1186/s12944-015-0093-3

**Published:** 2015-09-16

**Authors:** Paula Ramos Pinto, Débora Dias Ferraretto Moura Rocco, Ligia Shimabukuro Okuda, Adriana Machado-Lima, Gabriela Castilho, Karolline Santana da Silva, Diego Juvenal Gomes, Raphael de Souza Pinto, Rodrigo Tallada Iborra, Guilherme da Silva Ferreira, Edna Regina Nakandakare, Ubiratan Fabres Machado, Maria Lucia Cardillo Correa-Giannella, Sergio Catanozi, Marisa Passarelli

**Affiliations:** Lipids Laboratory (LIM - 10), University of São Paulo Medical School, Av. Dr. Arnaldo 455, room 3305, Sao Paulo, SP CEP 01246000 Brazil; Department of Physiology and Biophysics, Institute of Biomedical Sciences, University of Sao Paulo, São Paulo, Brazil; Laboratory of Endocrinology and Cellular Metabolism (LIM – 25), University of São Paulo Medical School, São Paulo, Brazil

**Keywords:** Atherosclerosis, HDL, Physical exercise, Reverse cholesterol transport, Macrophages

## Abstract

**Background:**

Regular exercise prevents and regresses atherosclerosis by improving lipid metabolism and antioxidant defenses. Exercise ameliorates the reverse cholesterol transport (RCT), an antiatherogenic system that drives cholesterol from arterial macrophages to the liver for excretion into bile and feces. In this study we analyzed the role of aerobic exercise on the *in vivo* RCT and expression of genes and proteins involved in lipid flux and inflammation in peritoneal macrophages, aortic arch and liver from wild type mice.

**Methods:**

Twelve-week-old male mice were divided into sedentary and trained groups. Exercise training was performed in a treadmill (15 m/min, 30 min/day, 5 days/week). Plasma lipids were determined by enzymatic methods and lipoprotein profile by fast protein liquid chromatography. After intraperitoneal injection of J774-macrophages the RCT was assessed by measuring the recovery of ^3^H-cholesterol in plasma, feces and liver. The expression of liver receptors was determined by immunoblot, macrophages and aortic mRNAs by qRT-PCR. ^14^C-cholesterol efflux mediated by apo A-I and HDL_2_ and the uptake of ^3^H-cholesteryl oleoyl ether (^3^H-COE)-acetylated-LDL were determined in macrophages isolated from sedentary and trained animals 48 h after the last exercise session.

**Results:**

Body weight, plasma lipids, lipoprotein profile, glucose and blood pressure were not modified by exercise training. A greater amount of ^3^H-cholesterol was recovered in plasma (24 h and 48 h) and liver (48 h) from trained animals in comparison to sedentary. No difference was found in ^3^H-cholesterol excreted in feces between trained and sedentary mice. The hepatic expression of scavenger receptor class B type I (SR-BI) and LDL receptor (B-E) was enhanced by exercise. We observed 2.8 and 1.7 fold rise, respectively, in LXR and *Cyp7a* mRNA in the liver of trained as compared to sedentary mice. Macrophage and aortic expression of genes involved in lipid efflux was not systematically changed by physical exercise. In agreement, ^14^C-cholestrol efflux and uptake of ^3^H-COE-acetylated-LDL by macrophages was similar between sedentary and trained animals.

**Conclusion:**

Aerobic exercise *in vivo* accelerates the traffic of cholesterol from macrophages to the liver contributing to prevention and regression of atherosclerosis, independently of changes in macrophage and aorta gene expression.

## Introduction

Regular physical exercise improves lipid metabolism, blood pressure, insulin sensitivity, endothelial function and haemostatic factors, reducing the incidence of coronary heart disease independently of other changes in life style [1–7]. In animal models of atherosclerosis it has been shown that aerobic exercise training reduces the area of pre established atherosclerotic lesions, ameliorates plaque stability and improves mice survival rate [8, 9]. These benefits can also be ascribed to the role of exercise in elevate antioxidant defenses in plasma and arterial wall, as well as, plasma HDL cholesterol levels.

HDL mediates the reverse cholesterol transport (RCT), an antiatherogenic system that drives cholesterol from the arterial wall to the liver assuring its excretion into feces. RCT is mediated by an orchestrated action of cholesteryl ester transfer protein (CETP), lipoprotein lipase, hepatic lipase, lecithin cholesterol acyltransferase (LCAT), ABC transporters (ABCA-1 and ABCG-1) and scavenger receptor class B, type I (SR-BI) [10].

By utilizing an *in vivo* measurement of the RCT, Meisser et al. (2010) did not demonstrate alteration in this system by utilizing a 2-week endurance voluntary exercise protocol [11]. Although, in the same animal model those authors (Meisser et al., 2010) found that a more prolonged exercise protocol enhanced biliary excretion of cholesterol which indicates a benefit in RCT [12]. Wei et al. (2005) showed enhanced expression of SR-BI and LDL-receptor mRNA levels in the liver of exercised mice, as an evidence for the improvement of the RCT by a 2-week aerobic exercise training protocol [13].

There is no evidence so far on the role played by aerobic exercise training - at the same volume/duration of that performed in studies showing atherosclerosis prevention or regression - in the RCT flow and in the expression of genes or levels of proteins that modulate this system. In this work, we analyzed the role of a 6-week well-controlled aerobic exercise training in the *in vivo* traffic of ^3^H-cholesterol from macrophages to plasma, liver and feces, the expression of genes and receptor levels involved in lipid flux in the liver, macrophages and aortic arch. The ability of macrophages isolated from trained and sedentary animals to export ^14^C-cholesterol to apo A-I or HDL_2_ as well as the uptake of ^3^H-cholesteryl oleoil ether acetylated LDL were also analyzed. We demonstrated that independently of changes in macrophage and aortic arch gene expression, aerobic training improves macrophage ^3^H-cholesterol flux to the liver. This was related to a greater amount of SR-BI protein level and *Cyp7a1* expression in the liver.

## Materials and methods

### Animals

C57BL/6N male mice (Taconic Inc, New York, USA) were fed regular chow *ad libitum* (Nuvilab-Nuvital, São Paulo, Brazil) with free access to water and were housed in conventional housing at 22 ± 2 °C with a 12 h light/12 h dark cycle. Animal experiments were performed in accordance with protocols approved by the Institutional Animal Care and Research Advisory Committee (Hospital das Clinicas of the University of São Paulo Medical School - CAPPesq # 773/06 and 441/11) and by the US National Institutes of Health Guidelines [14].

### Plasma lipid analysis

Cholesterol concentration in all lipoprotein fractions was measured by enzymatic colorimetric kit (Roche Diagnostic, Brazil). HDL-c was determined at the final period only by the ratio: HDL cholesterol area/total cholesterol area. Total plasma cholesterol and triglycerides were determined by enzymatic techniques (Labtest, Brazil) and glucose by Accu Check Performa glucometer (Roche, Brazil). Lipoprotein profile was determined by fast protein liquid chromatography (FPLC) gel filtration on two Superose 6 columns.

### Blood pressure measurement

Systolic blood pressure (BP) was assessed in conscious mice with a standard tail-cuff technique using an oscillometric method. Animals previously warmed for 12 min at 40°C were placed in a restrainer with the tail exiting through the rear hatch. BP measurements were considered only in the rested animal. After three successive days of mouse preconditioning to the measurement system, ten readings were carried out in two consecutive days and averaged to obtain mean values.

### Training protocol

Before starting training, animals from both groups, sedentary and trained were submitted to running adaptation. Briefly, animals were placed in a treadmill for 10 min at 8 m/min with a progressive increment up to 15 m/mim. Animals that were not able to keep running were excluded (8.1 % of exclusion).

Twelve-week-old animals were submitted to a 6-week monitored aerobic exercise training protocol performed on a treadmill (WEG CFW-08, São Carlos, Brazil) at 15 m/min during 30-min sessions, 5 times a week. A control group was kept sedentary during all period. Exercise sessions were carried out in the late afternoon. Animals reached 15 m/min after the first week of training animal at 12 m/min.

### Measurement of the *in vivo* RCT

J774 macrophages were incubated for 48 h in labeling media containing 50 μg/mL acetylated low-density lipoprotein (LDL) and 5 μCi/mL ^3^H-cholesterol. After 24 h incubation in DMEM containing fatty acid free albumin allowing the equilibratium of intracellular cholesterol pools, cells were washed, centrifuged and resuspended in PBS. Cell viability was superior to 98 % according to Trypan blue exclusion. Around 96 % of intracelular cholesterol was in free form as assessed by thin layer chromatography.

A hundred microliters of cell suspension (~3.2 × 10 ^6^ dpm) was injected into sedentary and trained mouse peritoneal cavity after 48 h of the last exercise session. Following, animals were individually housed in metabolic cages with free access to food and water to have blood drawn from the tail vein and feces collected 24 h and 48 h after ^3^H-cholesterol-labeled cells injection into peritoneal cavity.

Blood was centrifuged (1,500 rpm, 20 min, 4 °C) and the radioactivity determined. Forty eight hours after the injection, animals were euthanized and the liver, spleen, lung, heart, kidneys and adrenal glands removed. After washing with cold 0.9 % NaCl solution the organs were dried and weighed. Organs and feces were stored at −70° until processing. Briefly, they were mixed with a 2:1 (v:v) chloroform/methanol solution [15] and stored at 4 °C overnight for lipid extraction. The radioactivity was determined after lipid layer evaporation under nitrogen flow. The recuperation of ^3^H-cholesterol in plasma, organs and feces was expressed as percentages of total dose (dpm) injected per gram of sample (liver or feces) or plasma volume (mL). The recovery of radioactivity in spleen, lung, heart, kidney and adrenal glands was minimal (data not shown).

### Lipoproteins isolation and LDL acetylation and labelling

Procedures with humans were in accordance with the Declaration of Helsinki. All blood donors had signed an informed written consent form previously approved by The Ethical Committee for Human Research Protocols of the Hospital das Clinicas, University of São Paulo Medical School (CAPPesq # 773/06 and 441/11). Low density lipoprotein (LDL, d = 1,019 – 1,063 g/mL) and high density lipoprotein subfraction 2 (HDL_2_, d = 1,063 – 1,125 g/mL) were isolated from healthy plasma donors by sequential ultracentrifugation and further purified by discontinuous gradient ultracentrifugation. Protein content was determined by the Lowry procedure [16]. LDL acetylation was performed according to Basu et al. [17]. After extensive dialysis against ethylenediaminetetraacetic phosphate-buffered saline (EDTA-PBS), acetylated LDL (AcLDL) and HDL were maintained sterile at 4 °C under nitrogen atmosphere and used within a month. For some experiments LDL was labelled with ^3^H-cholesteryl oleoyl ether (^3^H-COE) according to Terpstra et al. [18].

### Western blotting analysis

Protein lysates were obtained by tissue homogenates in Polytron (MA099 Potter Unit, Marcone Equip., São Paulo, Brazil) by using buffer containing 20 mM Hepes, 150 mM NaCl, 10 % glycerol, 1 % triton, 1 mM EDTA, 1.5 mM MgCl_2_ and protease inhibitors. An aliquot of supernatant was obtained after centrifugation and dissolved in SDS-glycerol. Equal amounts of sample protein were applied into a polyacrylamide gel and immunoblotting performed for SR-BI, LXR and the LDL receptor by using anti-SR-BI 1:1,000, anti-LXR 1 :1,000 (Novus Biologicals, Inc., Littleton, CO, USA), and anti-LDL receptor 1:1,000 (Santa Cruz Biotechnology Inc, USA). Membranes were incubated with HRP-conjugated antibody and reacted against ECL (Super Signal West Pico Chemiluminescent substract, Pierce, Rockford, IL, EUA). Nitrocellulose membrane stripping was done by washing with NaOH 0.8 mM. The difference between the bands was analyzed in pixels using the JX-330 Color Image Scanner (Sharp®) and ImageMaster software (Pharmacia Biotech). Results are expressed as arbitrary units corrected per β-actin (anti β-actin 1:1,000, Fitzgerald Industries International, Inc., Concord, MA). β-actin was utilized as a control and Ponceau staining of nitrocellulose membranes was also implemented to assure equal protein loading.

### Gene expression analysis

Mice were euthanized in CO_2_ chamber and macrophages were harvested from peritoneal cavity immediately (0 h) or 48 h after the last session of exercise. Following, mice were transcardially perfused under low-pressure, with a 0.9 % NaCl cold solution and then, aortic arch and liver were excised in the fresh state and preserved in liquid nitrogen as far as analysis. RNA was isolated from tissues and macrophages by using Trizol (Invitrogen Life Technologies, Carlsbad, CA, USA). The cDNA was synthetized from 100 ng of total RNA using the High Capacity RNA-to-cDNA kit (Applied Biosystems). Real-time PCR was performed using Gene Expression Master Mix (Applied Biosystems). The following TaqMan Gene Expression Assays were used in the Step One Plus Real Time PCR System (Applied Biosystems): *Cyp7a1 -* Mm00484150_m1, *Cyp27a1 -* Mm00470430_m1, *Abca1* - Mm00442646_m1, *Abcg1* Mm00437390_m1, *Cd36* - Mm01135198_m1, *Olr1* Mm00454586_m1, *Scarb1* - Mm00450234_m1, *Pparg* - Mm01184322_m1, *Nr1h3* Mm01329744_g1, Nr1h2 - Mm00437265_g1, *Ccl2 -* Mm00441242_m1, *Tnf* - Mm00450234_m1, *Il6* -Mm00450234_m1, *Il10* - Mm00450234_m1. The relative expression of each gene was measured with respect to the expression of the housekeeping genes *Actb* - Mm00607939_s1 (macrophages and liver) and *Gapdh* – Mm99999915_g1 (aortic arch), which were used as endogenous reference to correct for differences in the amount of total RNA added to the reaction. The relative quantification of gene expression was performed with StepOne Software 2.0 (Applied Biosystems) using the comparative cycle threshold (Ct) (2^-ΔΔCt^) method [19, 20].

### Cholesterol efflux assay

Macrophages were harvested from the peritoneal cavities of sedentary and trained mice and placed in PBS containing 1 % penicillin-streptomycin and 4 mM L-glutamine. Cells were collected immediately (0 h) and 48 h after the last exercise bout. Cells were cultivated in RPMI containing 10 % fetal calf serum, 1 % penicillin-streptomycin and 4 mM L-glutamine, and they were maintained in a 5 % CO_2_ incubator at 37 °C for 24 h. After washing with PBS containing fatty acid free albumin (FAFA), cells were incubated with 50 μg/mL of acetylated LDL and 0.3 μCi/mL of ^14^C-cholesterol for 30 h. Cells were incubated with DMEM/FAFA for 24 h for equilibrate intracellular cholesterol pools and then incubated with 50 μg/mL of HDL_2−_protein or 30 μg of apo A-I as cholesterol acceptors. Purified human apo A-I was gently provided by Dr. Shinji Yokoyama from Nutritional Health Science Research Center, Chubu University. Control cells were incubated with DMEM/FAFA alone. Cholesterol efflux was determined after 8 h and 24 h. Medium was drawn and centrifuged at 1,500 rpm for 10 min to spin down cell debris, and the radioactivity was determined in the supernatant. Cell lipids were extracted three times with a hexane/isopropanol mixture (2:1;v:v), and the radioactivity was determined after solvent evaporation. Cell lysate was obtained after a two-hour incubation period with 0.2 N NaOH in order to measure protein concentration. The percentage of ^14^C-cholesterol efflux was calculated as (^14^C-cholesterol in the medium/^14^C-cholesterol in cells plus medium) × 100. The difference between the efflux elicited by HDL_2_ or apo A-I plus albumin and that by albumin-enriched media alone results in the HDL_2_ and A-I-mediated efflux.

### Uptake of acetylated LDL

Macrophages were incubated for 6 h in the presence of ^3^H-COE-acetylated LDL and LDL uptake (μg of LDL/mg of cell protein) calculated after cell washing, solubilization with 0.2 N NaOH, radioactivity counting and cell protein determination.

### Statistical analysis

Statistical analyses were performed using GraphPad Prism 5.0 software (GraphPad Prism, Inc., San Diego, CA). Unpaired Student’ *t* test was utilized to compare differences between groups. Summary data are reported as mean values ± standard error or mean values ± standard deviation as indicated. A p-value < 0.05 was considered statistically significant.

## Results

**Fig. 1:**
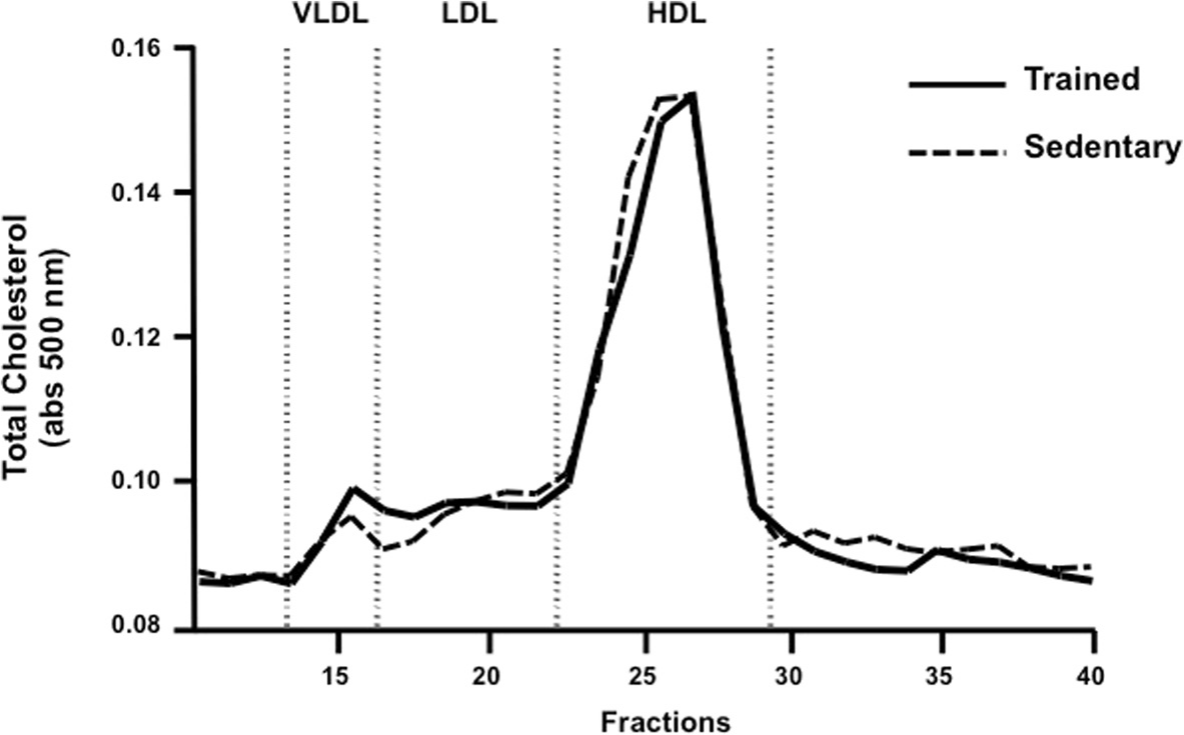
Plasma Lipoprotein profile of trained and sedentary mice after training protocol. Plasma lipoproteins were isolated by fast protein liquid chromatography (FPLC), and total cholesterol was determined in all fractions using an enzymatic kit. Trained (*filled line*) and sedentary (*dashed line*)

After six-week of aerobic exercise training, body weight, total plasma cholesterol, triglycerides and glucose concentration were not different between groups (Table 1). Similarly, plasma lipid profile assessed by FPLC was not changed by the aerobic exercise training (Fig. 1). Systolic blood pressure was reduced after exercise training (Table 1).

**Table 1:** Body weight, plasma lipids, glucose and blood pressure in trained and sedentary C57BL/6N mice

		Trained	Sedentary	p
		(*n* = 66)	(*n* = 69)	
Body weight (g)	Basal	26.1 ± 3.4	26.3 ± 3.4	0.736
	Final	27.4 ± 3.6	27.7 ± 4.5	0.645
Total cholesterol (mg/dL)	Basal	110 ± 14	107 ± 20	0.442
	Final	104 ± 19	109 ± 20	0.298
Triglycerides (mg/dL)	Basal	74 ± 19	70 ± 21	0.428
	Final	58 ± 16	58 ± 13	0.897
Glucose (mg/dL)	Basal	93 ± 20	95 ± 19	0.222
	Final	104 ± 25	106 ± 23	0.707
Blood pressure (mmHg)	Basal	84 ± 10	86 ± 5	0.222
	Final	76 ± 7	85 ± 7	0.007

Data are expressed as mean values ± standard deviation.

**Fig. 2:**
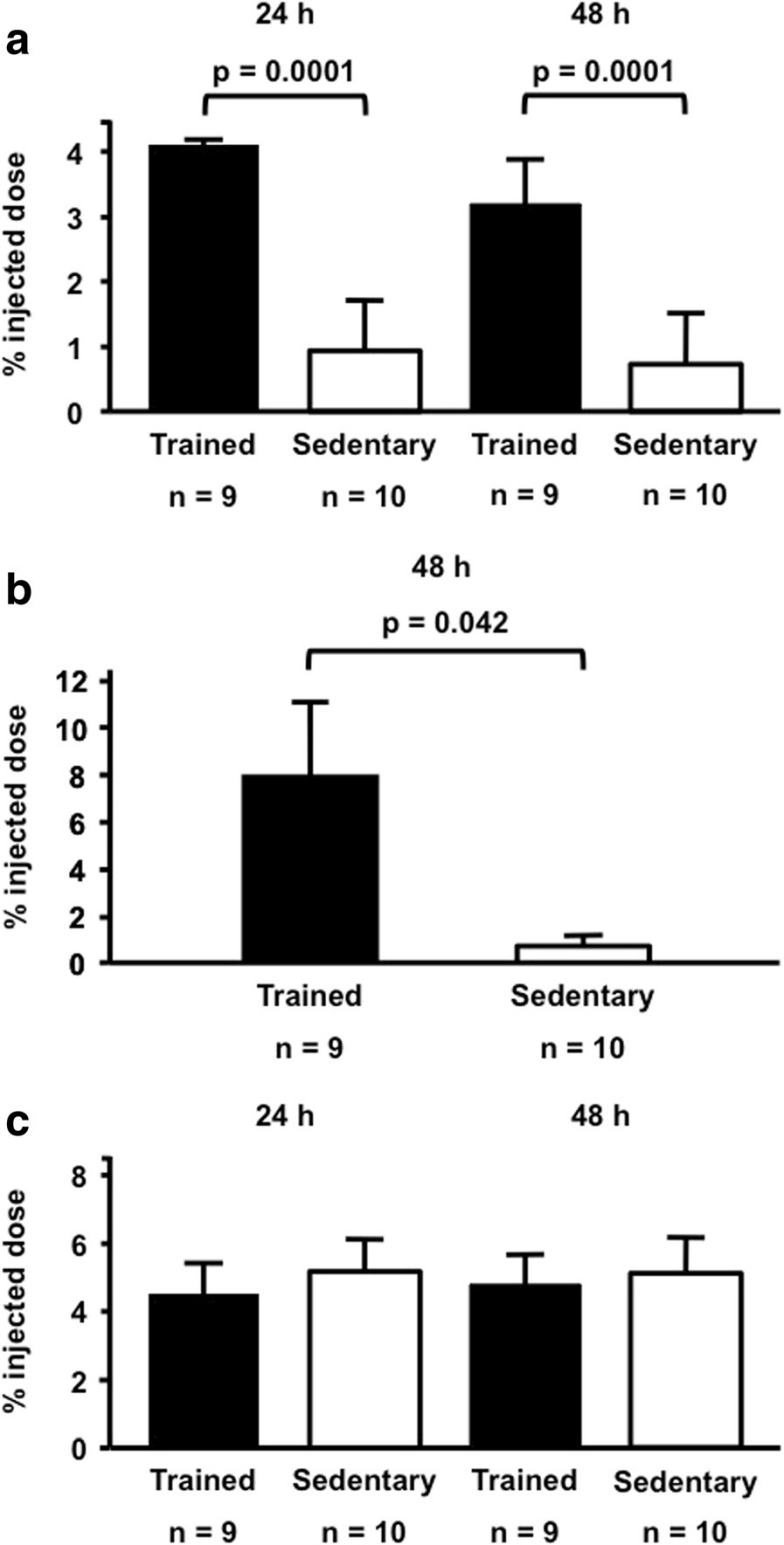
Recovery of ^3^H-cholesterol from intraperitoneal injected macrophages in: **a** plasma, **b** liver and **c** feces from aerobically trained and sedentary mice. J774 macrophages were enriched with acetylated LDL and ^3^H-cholesterol and injected into peritoneal cavity of trained and sedentary C57BL/6N wild type mice. The radioactivity was determined in plasma and feces after 24 h and 48 h and in the liver after 48 h. The recovery of ^3^H-cholesterol was expressed as the percentage of injected dose/mL of plasma or percentage of injected dose/mg of tissue or feces. Data are expressed as mean values ± standard error

The distribution of ^3^H-cholesterol was analyzed in plasma and feces at 24 h and 48 h and in the liver at 48 h following the intraperitoneal injection of J774 macrophages enriched with acetylated LDL and radiolabeled cholesterol. In the trained mice a higher amount of ^3^H-cholesterol was recovered in plasma and liver compared to the sedentary group (Fig. 2a and b). However, the amount of ^3^H-cholesterol excreted into feces was similar between trained and sedentary mice (Fig. 2c). Total fecal mass (g) was similar between trained and sedentary groups (respectively 24 h: 0.78 ± 0.1 *vs* 0.84 ± 0.3; 48 h: 0.8 ± 0.1 *vs* 1.0 ± 0.3).

**Fig. 3:**
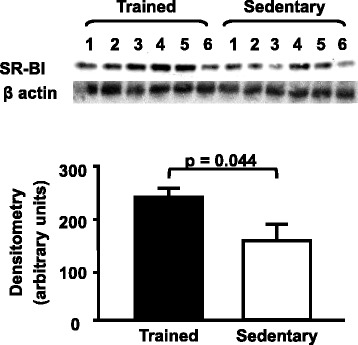
Hepatic SR-BI protein level in trained and sedentary C57BL/6N wild type mice. Equal amounts of liver lysates were applied into a 10 % polyacrylamide gel. Immunoblotting was performed by using an anti-SR-BI Ab (1:1000), incubation with secondary Ab conjugated with HRP and bands visualization after ECL reaction. Each lane represents one animal sample. Data are expressed as mean values ± standard error

**Fig. 4:**
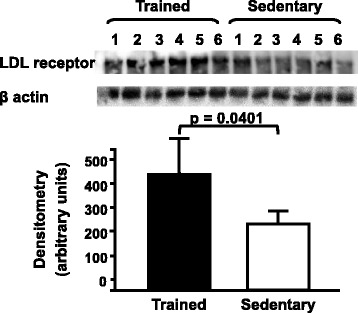
Hepatic LDL receptor protein level in trained and sedentary C57BL/6N wild type mice. Equal amounts of liver lysates were applied into a 10 % polyacrylamide gel. Immunoblotting was performed by using an anti-LDL receptor Ab (1:1000), incubation with secondary Ab conjugated with HRP and bands visualization after ECL reaction. Each lane represents one animal sample. Data are expressed as mean values ± standard error

**Fig. 5:**
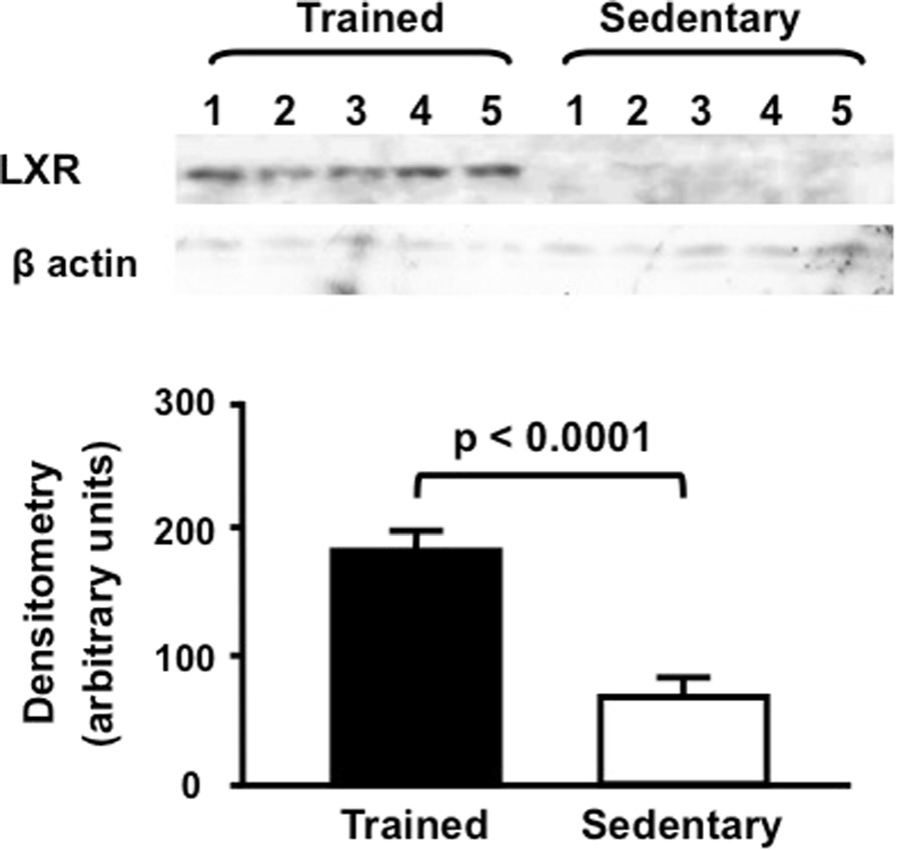
Hepatic LXR protein level in trained and sedentary C57BL/6N wild type mice. Equal amounts of liver lysates were applied into a 10 % polyacrylamide gel. Immunoblotting was performed by using an anti-LXR Ab (1:1000), incubation with secondary Ab conjugated with HRP and bands visualization after ECL reaction. Each lane represents one animal sample. Data are expressed as mean values ± standard error

**Fig. 6:**
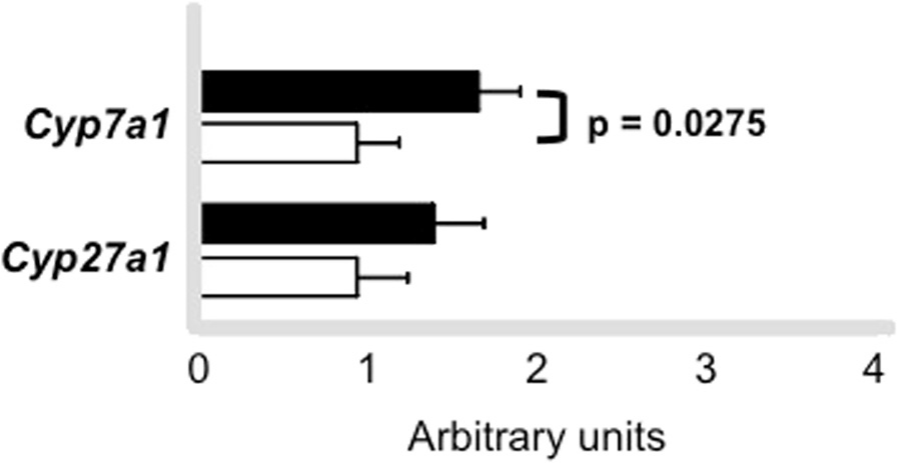
*Cyp7a1* and *Cy27a1* mRNA expression in the liver samples of trained and sedentary C57BL/6N wild type mice by quantitative real-time PCR. Using reverse transcriptase, cDNA was synthetized from 2 μg from total RNA isolated from the liver samples of trained (*n* = 6 - *black bars*) and sedentary (*n* = 6 - *white bars*). The TaqMan gene expression assays used were Mm00484150_m1 (*Cyp7a1*) and Mm00470430_m1 (*Cyp27a1*) and quantification was normalized to the endogenous *Actb* (Mm00607939_s1). Real-time PCR was performed using Gene Expression Master Mix (Applied Biosystems). Data analysis was performed using 2^-ΔΔCt^ method. Data are expressed as mean values ± standard error

The expression of SR-BI was 60 % enhanced in the liver of trained mice as compared to sedentary (Fig. 3). Also, a higher expression of hepatic LDL receptor was found in trained mice in comparison to sedentary animals (Fig. 4). Moreover, the expression of LXR was 2.8 fold elevated in the liver of trained compared to sedentary animals (Fig. 5). Aerobic exercise training raised the mRNA of *Cyp7A1* although no changes were observed in *Cyp27a1* mRNA expression (Fig. 6).

**Fig. 7:**
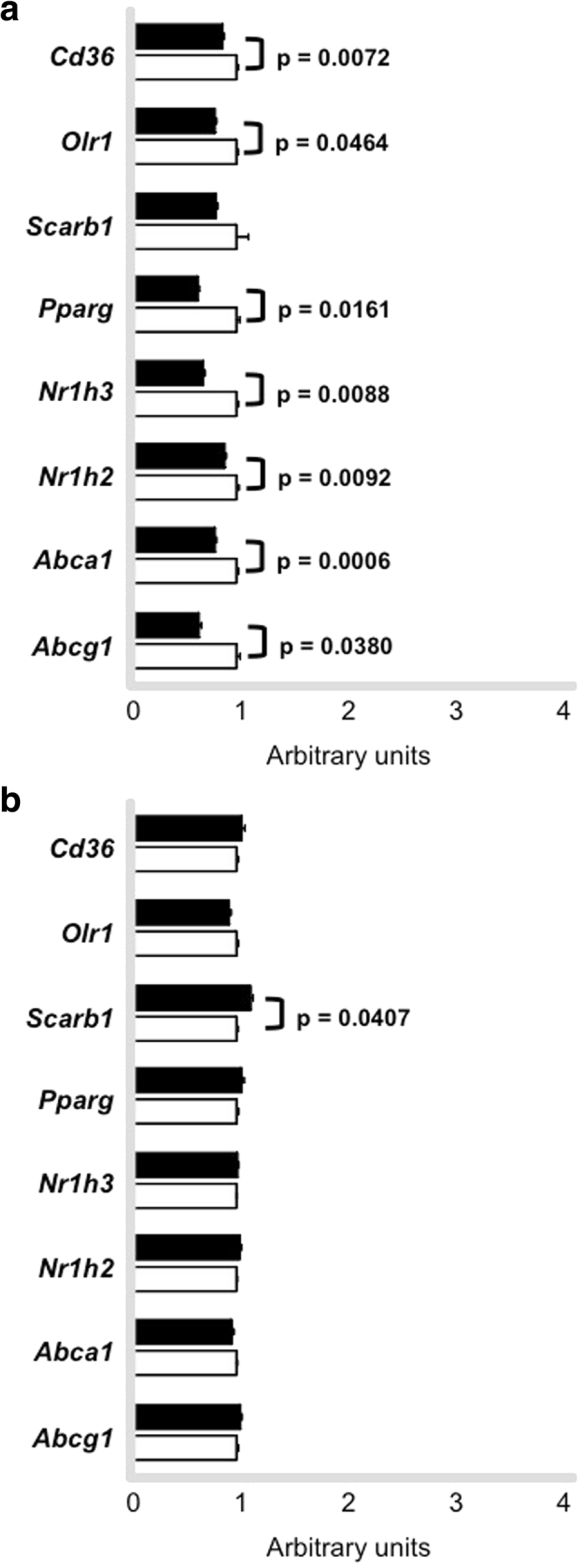
Expression of genes involved in lipid flux in macrophages. Peritoneal macrophages were harvested from trained (*n* = 6 - *black bars*) and sedentary (*n* = 6 *- white bars*) C57BL/6N wild type animals immediately (0 h **panel a**) and 48 h (**panel b**) after the last exercise session. Macrophages were ressuspendend in Trizol and gene expression was determined by quantitative real-time PCR. Using reverse transcriptase, cDNA was synthetized from 100 ng from total RNA isolated from macrophages of trained (*black bars*) and sedentary (*white bars*). The TaqMan gene expression assays used were: *Cd36* - Mm01135198_m1, *Olr1* Mm00454586_m1, *Scarb1* - Mm00450234_m1, *Pparg* - Mm01184322_m1, *Nr1h3* Mm01329744_g1, *Nr1h2* - Mm00437265_g1, *Abca1* - Mm00442646_m1, *Abcg1* Mm00437390_m1 and quantification was normalized to the endogenous *Actb* (Mm00607939_s1). Real-time PCR was performed using Gene Expression Master Mix (Applied Biosystems). Data analysis was performed using 2^-ΔΔCt^ method. Data are expressed as mean values ± standard error

Peritoneal macrophages were isolated in order to investigate acute and chronic effects of exercise in gene expression. Among genes involved in LDL uptake by macrophages, *Cd36* and *Orl1* mRNA presented reduced levels at time 0 h in trained animals as compared to sedentary although no changes were further observed at 48 h. On the contrary, *Scarb1* mRNA levels were only enhanced in cells isolated after 48 h of exercise session. Genes related to cholesterol exportation to apo A-I and HDL_2_, respectively, *Abca1* and *Abcg1* were acutely reduced by exercise (0 h) although changes were no longer observed in cells isolated after 48 h. In agreement, transcriptional factors mRNA that modulate HDL receptors, such as *Pparg*, *Nr1h3* and *Nr1h2* were also reduced in macrophages isolated from trained animals immediately after the exercise session in comparison to sedentary animals. In cells isolated after 48 h no changes were observed between trained and sedentary groups (Fig. 7a and b).

**Fig. 8:**
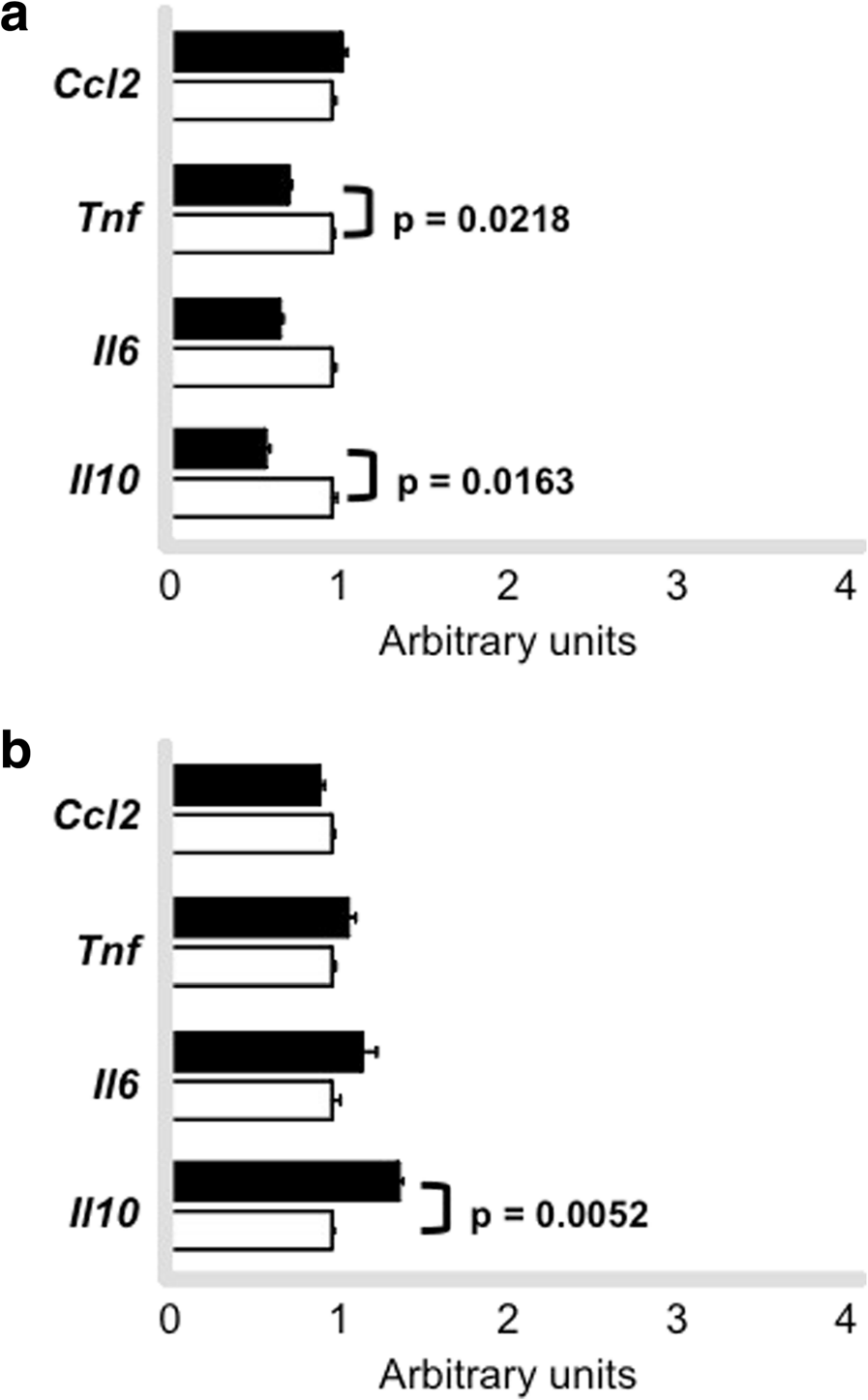
Expression of genes involved in inflammatory response in macrophages. Peritoneal macrophages were harvested from trained (*n* = 6) and sedentary (*n* = 6) C57BL/6N wild type animals immediately (0 h - **panel a**) and 48 h (**panel b**) after the last exercise session. Macrophages were ressuspended in Trizol and gene expression was determined by quantitative real-time PCR. Using reverse transcriptase, cDNA was synthetized from 100 ng from total RNA isolated from macrophages of trained (*black bars*) and sedentary (*white bars*). The TaqMan gene expression assays used were: *Ccl2 -* Mm00441242_m1, *Tnf* - Mm00450234_m1, *Il6* -Mm00450234_m1, *Il10* - Mm00450234_m1 and quantification was normalized to the endogenous *Actb* (Mm00607939_s1). Real-time PCR was performed using Gene Expression Master Mix (Applied Biosystems). Data analysis was performed using 2^-ΔΔCt^ method. Data are expressed as mean values ± standard error

We also measured mRNA levels of genes that mediate inflammatory response that is known to modulate macrophage lipid metabolism. *Tnf* and *Il10* mRNAs were reduced in macrophages after exercise session (0 h) in trained animals as compared to sedentary mice. On the other hand, *Il10* mRNA was increased in cells isolated after 48 h exercise session. *Ccl2* and *Il6* were not changed when comparing sedentary and trained groups in cells isolated at 0 h and 48 h (Fig. 8a and b).

**Fig. 9:**
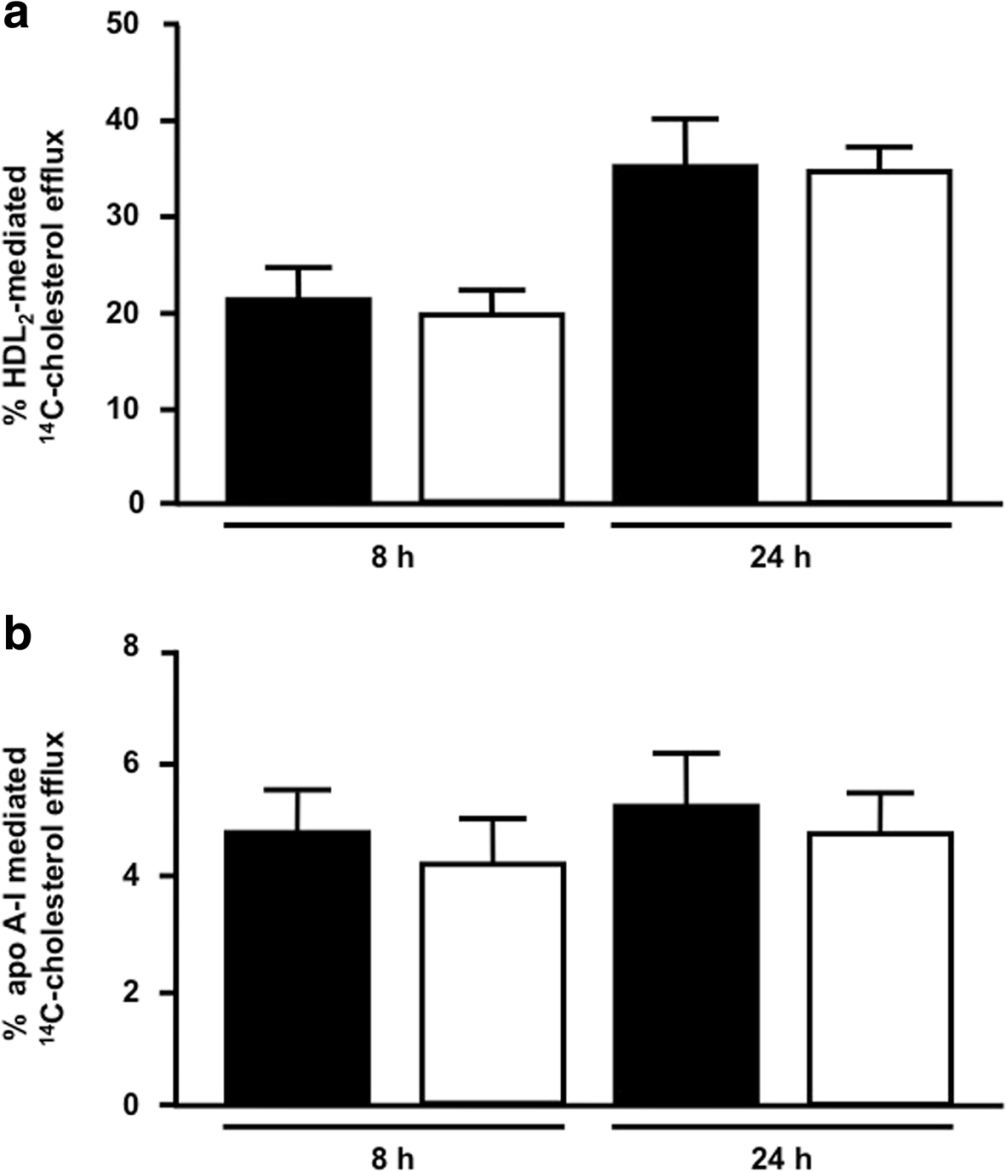
HDL_2_ and apo A-I mediated ^14^C-cholesterol efflux from peritoneal macrophages. Macrophages isolated from peritoneal cavity of trained (*n* = 7 - *black bars*) and sedentary (*n* = 7 - *white bars*) C57BL/6N wild type mice, after 48 h the last exercise session, were enriched with acetylated LDL and ^14^C-cholesterol 30 h. The ^14^C-cholesterol efflux was determined, after 8 h and 24 h, of incubation with HDL_2_ (**panel a**) and apo A-I (**panel b**). ^14^C-cholesterol efflux was calculated as (^14^C-cholesterol in the medium/^14^C-cholesterol in cells plus medium) × 100. Data are expressed as mean values ± standard deviation

**Fig. 10:**
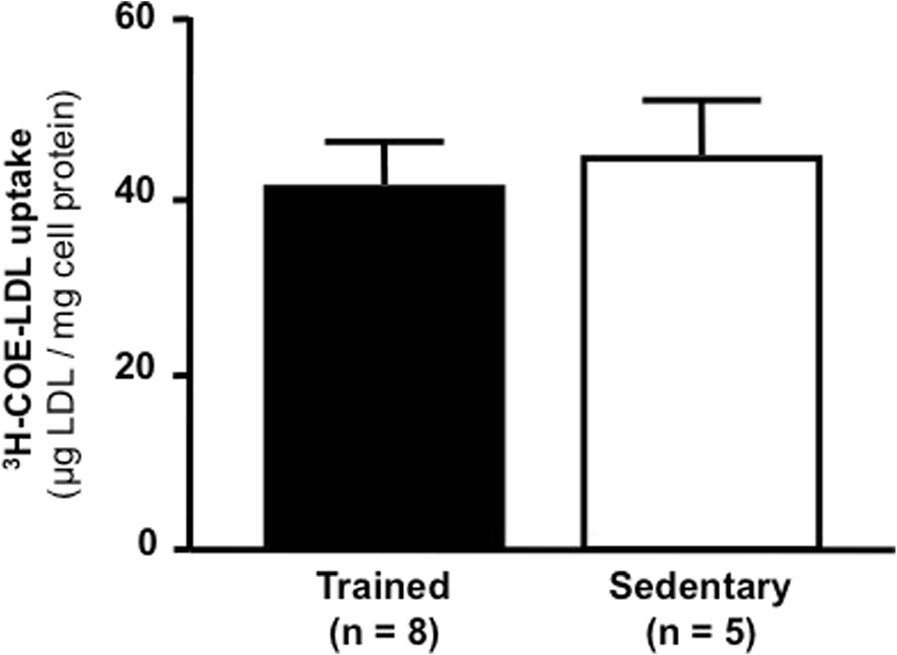
Uptake of ^3^H-COE-acetylated LDL by peritoneal macrophages. Macrophages isolated from trained (*n* = 8) and sedentary (*n* = 8) C57BL/6N wild type mice, were incubated with ^3^H-COE-acetylated LDL for 6 h. The uptake was calculated by radioactivity counting and cell protein determination. Data are expressed as mean values ± standard deviation

The ability of peritoneal macrophages isolated from sedentary and trained animals, after 48 h of the last exercise session, to export cholesterol to HDL_2_ or apo A-I was analyzed in *in vitro* incubations. The apo A-I and HDL_2_-mediated cholesterol efflux (8 h and 24 h) from macrophages was similar between groups (Fig. 9). Similarly, the uptake of ^3^H-COE by these cells was not changed by exercise (Fig. 10).

**Fig. 11:**
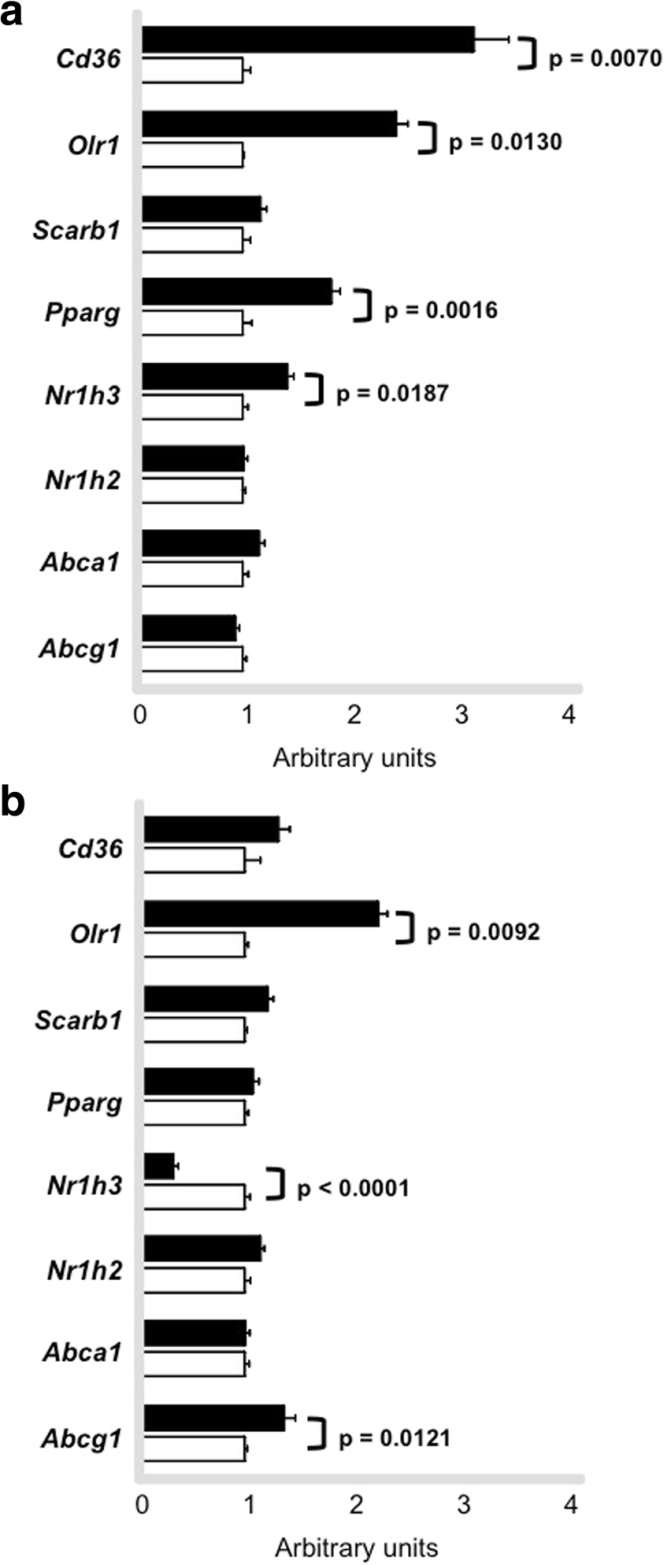
Expression of genes involved in lipid flux in aorta. Aortic arch was isolated from trained (*n* = 7) and sedentary (*n* = 6) C57BL/6N wild type mice immediately (0 h - **panel a** ) and 48 h (**panel b**) after the last exercise session. Gene expression was determined by quantitative real-time PCR. Using reverse transcriptase, cDNA was synthetized from 100 ng from total RNA isolated from aortic arch of trained (*black bars*) and sedentary (*white bars*). The TaqMan gene expression assays used were: *Cd36* - Mm01135198_m1, *Olr1* Mm00454586_m1, *Scarb1* - Mm00450234_m1, *Pparg* - Mm01184322_m1, *Nr1h3* Mm01329744_g1, *Nr1h2* - Mm00437265_g1, *Abca1* - Mm00442646_m1, *Abcg1* Mm00437390_m1 and quantification was normalized to the endogenous *Gapdh* (Mm99999915_g1). Real-time PCR was performed using Gene Expression Master Mix (Applied Biosystems). Data analysis was performed using 2^-ΔΔCt^ method. Data are expressed as mean values ± standard error

In aorta, as opposed to peritoneal macrophages, *Cd36* and *Orl1* mRNA were elevated at time 0 h but only *Orl1* was kept high at 48 h. *Scarb1* mRNA levels were not changed between groups in both periods of aortic arch isolation. *Abca1* and *Abcg1* were not changed in aortas immediately isolated after exercise when comparing sedentary and trained groups. *Abcg1* mRNA was increased in aortic tissue isolated after 48 h of exercise bout in trained animals. *Pparg* and *Nr1h3* mRNA were acutely elevated in cells by exercise in trained animals, although *Nr1h3* was decreased in macrophages at 48 h (Fig. 11).

**Fig. 12:**
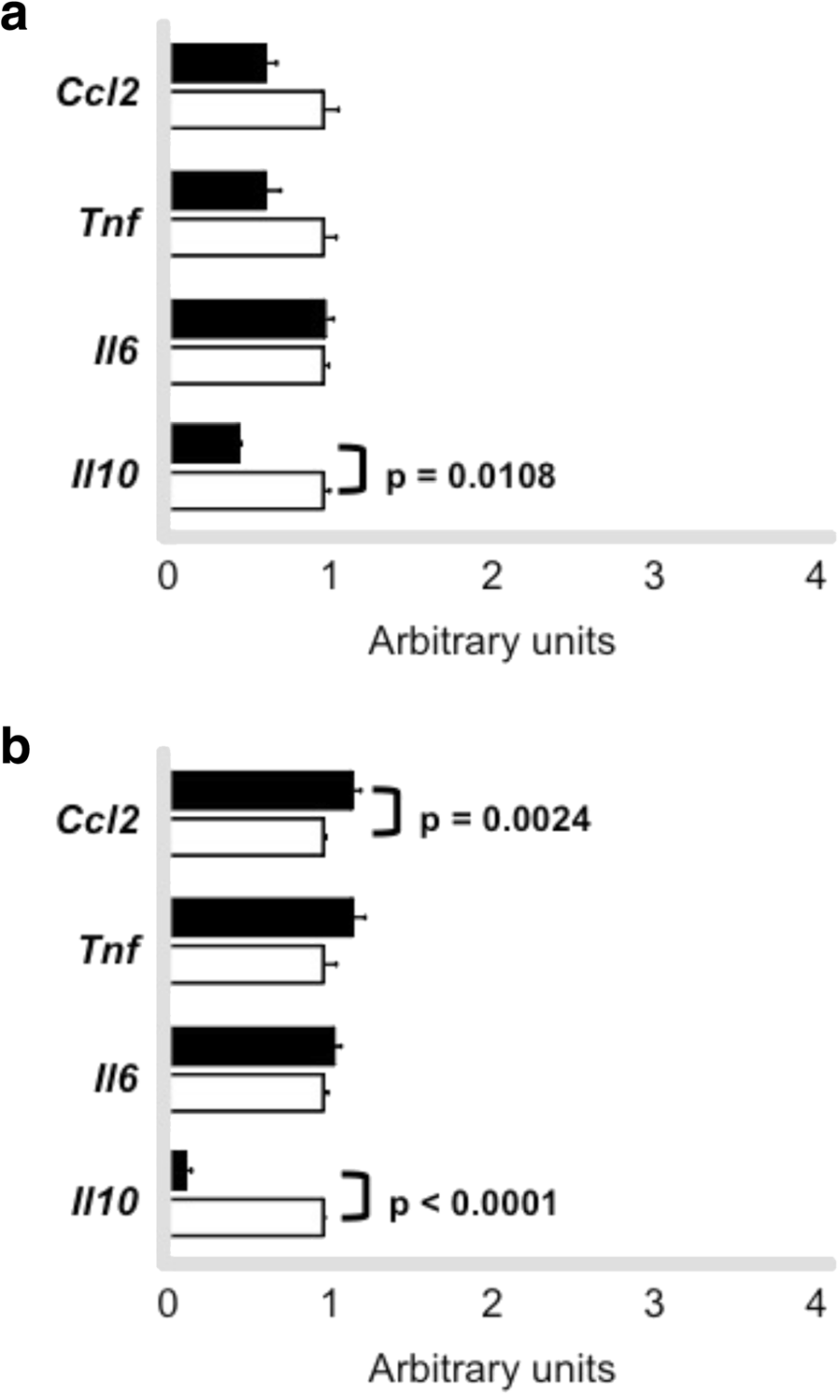
Expression of genes involved in inflammatory response in aorta. Aortic arch was isolated from trained (*n* = 7) and sedentary (*n* = 7) C57BL/6N wild type mice immediately (0 h - **panel a**) and 48 h (**panel b**) after the last exercise session. Gene expression was determined by quantitative real-time PCR. Using reverse transcriptase, cDNA was synthetized from 100 ng from total RNA isolated from aortic arch of trained (*black bars*) and sedentary (*white bars*). The TaqMan gene expression assays used were: *Ccl2 -* Mm00441242_m1, *Tnf* - Mm00450234_m1, *Il6* -Mm00450234_m1, *Il10* - Mm00450234_m1 and quantification was normalized to the endogenous *Gapdh* (Mm99999915_g1). Real-time PCR was performed using Gene Expression Master Mix (Applied Biosystems). Data analysis was performed using 2^-ΔΔCt^ method. Data are expressed as mean values ± standard error

*Tnf* and *IL6* mRNA levels were similar between sedentary and trained mice in both periods of aorta isolation. *Il10* expression was decreased in trained mice in both periods (0 h and 48 h) and *Ccl2* was increased only in aorta isolated after 48 h of exercise session compared to sedentary animals (Fig. 12).

## Discussion

Regular physical exercise improves lipid metabolism and contributes to the prevention of atherosclerosis. In this study, we investigated in wild type C57BL6N mice the role of a six-week aerobic exercise training program on the *in vivo* RCT and gene expression in peritoneal macrophages and aortic arch. Our results demonstrated that exercise training improves the recovery of ^3^H-cholesterol from peritoneal macrophages in plasma and liver, enhanced the hepatic expression of SR-BI, LXR and B-E receptor protein and increased the mRNA of *Cyp7a1* in the hepatic tissue, independently of changes in gene expression in macrophages and aorta.

Wei C et al. (2005) demonstrated that 2 weeks of aerobic exercise raises the mRNA of hepatic SR-BI in mice although in that paper authors did not determine the final protein content in the animal’s liver [13]. SR-BI is known as an important regulator of the final step of the RCT, since it drives cholesterol for excretion into bile. In fact, SR-BI knockout mice besides having higher levels of plasma HDL cholesterol develop atherosclerosis [21]. On the other hand, SR-BI overexpression protects mice against diet-induced atherosclerosis despite of low HDL plasma levels [22]. In humans, SR-BI mutations lead to the impairment in its activity although has not been related to alteration in carotid intima-media thickness [23]. In our study the enhanced level of SR-BI expression contributed to a higher amount of ^3^H-cholesterol recovered in the liver of trained animals as compared to sedentary mice. Besides, the enhanced expression of this receptor may have masked the exercise-induced elevation in HDL cholesterol that has been described by others in rats and by our group in CETP-tg mice [24–26].

In accordance with previous data from literature [13, 27] we have shown an increased expression of hepatic LDL receptor (B-E). Nonetheless, in our animal model this receptor does not contribute to the last step of the RCT due to the absence of CETP.

In addition, the benefit of exercise training to the RCT was reflected by the elevated expression of *Cyp7a1* mRNA, enzyme that converts free cholesterol into cholic acid the major route of bile acids synthesis. Surprisingly, we did not find differences in the ^3^H-cholesterol excretion into feces which may be ascribed to the experimental time points utilized (24 h and 48 h after ^3^H-cholesterol-labeled J-774 foam cells injection into peritoneal cavity). Also, we did not measure the *Abcg5/8* expression and excretion of bile acids and neutral lipids in feces which is a limitation of our study. In this regard, a recent investigation [28] demonstrated that in wild type mice, 12 weeks of voluntary running wheel modulated cholesterol catabolism by enhancing biliary bile acid secretion and increased fecal bile acid and neutral sterol outputs compared to sedentary controls.

In human CETP transgenic (CETP-tg) mice we previously showed [26] that aerobic exercise training improved RCT by increasing the recovery rate of macrophage ^3^H-cholesterol injected into peritoneal cavity in plasma and liver. Additionally, in this model we also found a higher amount of ^3^H-cholesterol in feces, completing the last step of the RCT. There were no changes in hepatic SR-BI content although a huge elevation was observed in B-E receptor protein level, bypassing cholesterol flux to the liver by the uptake of apo B-containing lipoproteins. In addition, compared to sedentary animals, trained CETP-tg mice presented higher levels of HDL-cholesterol in plasma and a higher ABCA-1 content in the liver. These events were not observed in WT mice in the present investigation that presented similar levels of HDL cholesterol and no changes in the ABCA-1 protein levels. Together with the enhancement in B-E receptor, this may explain why in CETP-tg mice we were able to observe an elevation in cholesterol excretion in feces that was not found in WT mice.

The expression of LXR a nuclear receptor that modulates the transcription of several genes involved in lipid metabolism, was increased by exercise in WT mice, although surprisingly we could not detect ABCA-1 in the liver of trained and sedentary mice.

A higher transference of radiolabed cholesterol from macrophages to plasma of trained animals observed by us in the *in vivo* analyses of the RCT cannot be attributed to enhancement in the cholesterol efflux rate. In fact, no changes were observed in cholesterol efflux in macrophages isolated after 48 h of exercise session in trained as compared to sedentary animals. It is noteworthy that the expression of *Abca1, Abcg1, Pparg*, *Nr1h3* and *Nr1h2* mRNA levels were acutely reduced by an exercise session but not after 48 h of exercise agreeing with the results of cholesterol exportation to apo A-I and HDL_2_. *Scarb1* increased in macrophages from trained mice, although it did not interfere in cholesterol removal, considering that in macrophages overloaded with sterols, ABCA-1 is responsible for the major amount of cholesterol efflux to apo A-I. In addition, the alterations in inflammatory genes elicited by exercise did not affect cell cholesterol removal. In aorta, we did not find systematic changes in the expression of genes that modulate lipid flux, except for *Abcg1* at time 48 h, suggesting that benefits elicited by exercise in the arterial wall site may not be totally related to the local modulation of RCT mediators.

The apparent discrepancy between the in vivo and in vitro experiments is probably related to the interplay of several components of the RCT that take place in vivo, including HDL levels, LCAT, lipoprotein lipase and hepatic lipase activities and receptors and enzymes that help to drive cholesterol to the liver. The *in vitro* experiments were designed in order to exclusively reflect possible cell changes induced by exercise in cell compartment. In that case, the concentration of HDL or apo A-I and physico-chemical properties of these particles were unlikely to influence cell cholesterol removal. On the other hand, these variables were present in the *in vivo* experiments helping to drive cholesterol to the liver apart from specific changes in macrophage gene expression.

In conclusion, aerobic exercise training improves the cholesterol trafficking from macrophages to the liver in WT mice, which is related to the enhancement in hepatic SR-BI protein level together with a higher expression of *Cyp7a1* and LXR, independently of systematic changes in macrophage and aorta gene expression. From the point of view of the RCT, the benefits of regular exercise in preventing atherosclerosis can be ascribed to an interplay of actions on systemic modulators of this transport, including HDL, and on the expression of hepatic and intestinal receptors that help to drive cholesterol from peripheral cell for excretion into feces.
